# The role of n plant glycosylation in Act d 2 allergenicity

**DOI:** 10.1186/2045-7022-4-S2-P16

**Published:** 2014-03-17

**Authors:** Araceli Diaz-Perales, Maria Garrido-Arandia, Amaya Murua-Garcia, Leticia Tordesillas, Cristina Garcia-Casado, Natalia Blanca-López, Tania Ramos, Gabriela Canto, Carlos Blanco, Javier Cuesta-Herranz, Luis F Pacios, Rosa Sanchez-Monge

**Affiliations:** 1 Technical University of Madrid, Center for Plant Biotechnology and Genomic, Campus de Montegancedo., Pozuelo de Alarcon, Madrid, 28, Spain; 2 Technical University of Madrid, Center for Plant Biotechnology, Pozuelo de Alarcon, Madrid, Spain; 3 Infanta Leonor Hospital, Allergy Service, Madrid, Spain; 4 La Princesa Hospital, Allergy Service, Madrid, Spain; 5 Jimenez Diaz Foundation, Allergy Service, Madrid, Spain

## Background

Plant allergens have hitherto been included in only several protein families that share no common biochemical features. Their physical, biochemical and immunological characteristics have been widely studied, but no definite conclusion has been reached about what makes a protein an allergen. N-glycosylation is characteristic of plant allergen sources but is not present in mammals.

## Objective

To evaluate and to compare the allergenic activity of the protein fraction and the N-glycan fraction of the thaumatin-like protein (TLP) and the main kiwi allergen, Act d 2.

## Methods

The natural allergen, Act d 2, was deglycosylated by TMSF treatment; the N-glycan fraction was obtained by extended treatment with proteinase K. The comparison of allergenic activity was carried out by immunoblot, ELISA and basophil activation assays. The ability to activate immune system cells was measured by T lymphocyte activation and monocyte-derived dendritic cell maturation.

## Results

N-glycan and protein fractions were recognized by specific IgE of kiwi-allergic patients. By contrast, the sugar moiety showed a reduced capacity to activate basophils and T cells, but not dentritic cells derived of patient's monocytes. In this sense, the proinflammatory cytokine production, measured as IL6 and IL10, was increased by the incubation of dendritic cells with the sugar moiety.

## Conclusions

The sugar moiety plays a significant role in sensitization, inducing the activation of antigen presenting cells. Nevertheless, the protein fraction is the responsible for the allergic reactions.

